# Highly UV Resistant Inch‐Scale Hybrid Perovskite Quantum Dot Papers

**DOI:** 10.1002/advs.201902439

**Published:** 2020-07-24

**Authors:** Ting‐You Li, Xuezhu Xu, Chun‐Ho Lin, Xinwei Guan, Wei‐Hao Hsu, Meng‐Lin Tsai, Xiaosheng Fang, Tom Wu, Jr‐Hau He

**Affiliations:** ^1^ Computer, Electrical, and Mathematical Sciences and Engineering (CEMSE) Division King Abdullah University of Science and Technology (KAUST) Thuwal 23955‐6900 Saudi Arabia; ^2^ School of Materials Science and Engineering University of New South Wales (UNSW) Sydney NSW 2052 Australia; ^3^ Institute of Physics Academia Sinica Nankang Taipei 115 Taiwan; ^4^ Department of Materials Science and Engineering National Taiwan University of Science and Technology Taipei 106 Taiwan; ^5^ Department of Materials Science Fudan University Shanghai 200433 P. R. China; ^6^ Department of Materials Science and Engineering City University of Hong Kong Hong Kong SAR 999077 China

**Keywords:** cellulose nanocrystals, displays, light‐emitting diodes, papers, perovskites, quantum dots, solar cells

## Abstract

Halide perovskite quantum dots (PQDs) are promising materials for diverse applications including displays, light‐emitting diodes, and solar cells due to their intriguing properties such as tunable bandgap, high photoluminescence quantum yield, high absorbance, and narrow emission peaks. Despite the prosperous achievements over the past several years, PQDs face severe challenges in terms of stability under different circumstances. Currently, researchers have overcome part of the stability problem, making PQDs sustainable in water, oxygen, and polar solvents for long‐term use. However, halide PQDs are easily degraded under continuous irradiation, which significantly limits their potential for conventional applications. In this study, an oleic acid/oleylamine (traditional surface ligands)‐free method to fabricate perovskite quantum dot papers (PQDP) is developed by adding cellulose nanocrystals as long‐chain binding ligands that stabilize the PQD structure. As a result, the relative photoluminescence intensity of PQDP remains over ≈90% under continuous ultraviolet (UV, 16 W) irradiation for 2 months, showing negligible photodegradation. This proposed method paves the way for the fabrication of ultrastable PQDs and the future development of related applications.

## Graphical abstract

Solid‐state perovskite quantum dot papers are fabricated using a unique vacuum filtration growth method without a purification process. The bonding between cellulose nanocrystals and perovskite quantum dots makes the hybrid structure stable, and record high UV stability and thermal stability are achieved for perovskite quantum dot papers.

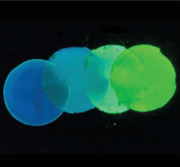

Perovskite quantum dots (PQDs) have been widely studied for developing the next‐generation display technology such as quantum dot (QD) enhancement films, QD light‐emitting diodes, and QD color filters over the past several years due to their inexpensive, color‐tunable, narrow emission peak (<20 nm), cadmium‐free, and high photoluminescence quantum yield (PLQY > 95%) properties.^[^ ^1^, ^2^, ^3^, ^4^, ^5^, ^6^, ^7^, ^8^, ^9^, ^10^, ^11^, ^12^, ^13^, ^14^, ^15^, ^16^, ^17^ ^]^ However, PQDs can be susceptible to degradation under H_2_O and O_2_‐rich environment, continuous irradiation condition, or even in polar solvent due to their intrinsically unstable properties.^[^ ^18^, ^19^, ^20^, ^21^, ^22^, ^23^, ^24^, ^25^, ^26^, ^27^, ^28^, ^29^ ^]^ In order to solve the problem, strategies have been provided including the adoption of alkyl ammonium with dozen‐nanometer chains and the most commonly used mixture of carboxylic acid (such as oleic acid (OA)) and alkylamines (such as oleylamine (OLA)) and alkylamines (such as oleylamine, OLA) in the perovskite precursor for stabilizing the PQDs in colloidal form.^[^ ^9^, ^10^ ^]^ In 2016, solid‐state PQDs were stabilized via surface protection of polyhedral oligomeric silsesquioxane to exhibit water‐resistant behavior.^[^ ^21^ ^]^ Other methods such as using mesoporous silica/PQD core‐shell structures have also been demonstrated to prevent photooxidation and show the improved relative intensity after 96 h continuous ultraviolet (UV) irradiation from 60% to 80%.^[^ ^20^ ^]^ In the above methods, the protective encapsulation has usually been applied after the synthesis of PQD. Therefore, surface ligands such as OA and OLA should still be used during the synthesis stage for controlling the size of the PQD and passivating the PQD structure before the encapsulation. Although encapsulation strategies during synthesis have also been carried out to improve water and thermal resistances of PQDs, the stability under continuous irradiation has not yet evaluated.^[^ ^30^ ^]^

In this study, instead of mixing carboxylic acid (such as OA) and alkylamines (such as OLA) in perovskite precursor, we used the cellulose nanocrystal (CNC) with a few hundred nanometers in length which contains a large amount of ‐HSO_3_^−^ and ‐O^−^ group to synthesize the perovskite quantum dot paper (PQDP). These ‐HSO_3_^−^ and ‐O^−^ cellulose anions work as the capping ligands which limit the growth of perovskite crystals and become PQDs well‐dispersed in PQDP. In addition, we propose that the ‐O^−^ and ‐HSO_3_^−^ of the CNCs are coordinated to PbX_2_ empty orbital during the preparation of perovskite precursor (*N*,*N*‐dimethylformide (DMF) + MAX + PbX_2_ + CNCs, MA = CH_3_NH_3_^+^), and the PQDs are entangled between long chains of CNCs to avoid the dangling capping behavior of OA and OLA (which may be easily destroyed under high polar solvents) and become more stable.^[^ ^31^ ^]^

Additionally, we conceived a unique method of vacuum filtration growth of PQD in this study. For conventional PQD synthesis, the growth of PQD is in the colloidal solution, and the resulting PQD product is in the solution form, which requires purification procedure and spin coating to convert into solid. In the vacuum growth method, the perovskite precursor and CNC mixed solution is vacuum‐filtered to remove the DMF solvent, and the precursor/CNC residues are collected on the filter membrane. After the solvent is removed, the precursors start to crystalize into perovskite QDs with the assistance of surrounding CNC capping ligands, resulting in solid‐state PQDP. Moreover, the PQDs are confined spatially and protected in the entangled cellulose structure during the synthesis, which eliminates the need of additional encapsulation process. The differences between conventional solution growth and vacuum filtration growth are shown in Table S1, Supporting Information. The stability of PQDP was tested under different circumstances. The relative photoluminescence (PL) intensity of PQDP remains at ≈80% after exposed in an environment of 60% relative humidity (RH) and 20 °C for 8 months and ≈90% after exposed in continuous 16 W UV irradiation for 1000 h, showing unprecedentedly high stability. With superior optoelectronic properties and photostability, the PQDP reported in this work is a favorable choice for next‐generation lighting and solar‐related applications.

CNCs are needlelike nanocrystals of 10–20 nm in width and several hundred nanometers in length, which are produced from various biological sources (e.g., bleached wood pulp, cotton, manila, tunicin, bacteria, etc.) by strong acid hydrolysis.^[^ ^32^, ^33^, ^34^, ^35^, ^36^ ^]^ They are composed of linear polysaccharide chains consisting of repeated *β*‐(1→4)‐d‐glucopyranose units. Owing to the presence of abundant hydroxyl (—OH) and sulfate (—OSO_3_Na) groups, CNCs are expected to exhibit a high electronegativity to complex with cations, that is, CH_3_NH_3_^+^, Pb^2+^ in metallic halide perovskites (**Figure** **1**a).

**Figure 1:**
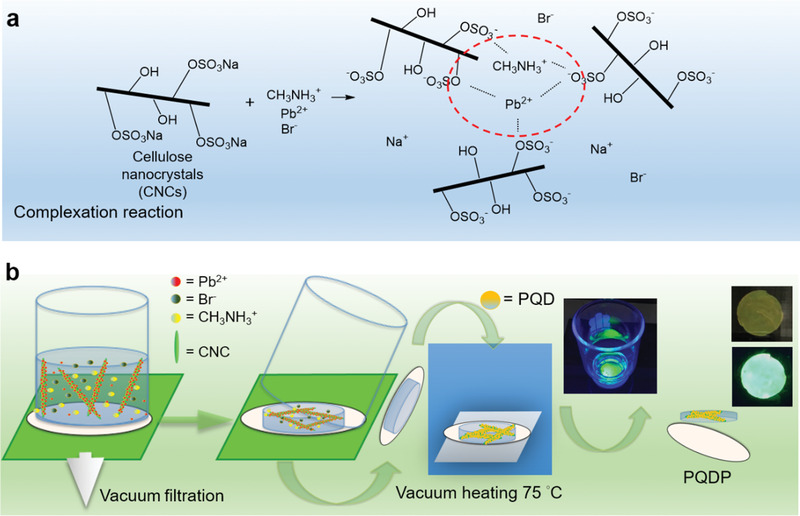
Illustration of the fabrication process. a) Proposed complexation reaction between CNCs and anions in perovskite. b) Preparation flowchart from CNC/DMF and metallic‐halide perovskite precursors to PQDP.

The PQDPs were produced using vacuum filtration followed by vacuum‐assisted heating at 75 °C.^[^ ^37^ ^]^ As sketched in Figure 1b, CNCs with predetermined weight ratios of PbBr_2_ and MABr dissolved in DMF solution were mixed by stirring for 30 min (250 rpm, RT), and the concentration of CNC was kept constant at 0.5 wt%. The mixed solution was first vacuum‐filtered using an Anopore membrane (0.02 µm pore size, Whatman International Ltd.) in a glass funnel (Wheaton) to remove unwanted DMF solvent, and the precursor/CNC mixtures were collected on the membrane. Then, the mixtures and membrane were taken from the funnel, followed by vacuum drying in a vacuum oven under the pressure of 0.36 kPa at 75 °C for 1 h to form the PQDP. Finally, the PQDP was peeled off from the membrane. Similar to commonly used papers, the resulting solid‐state PQDP is flexible and robust. Moreover, the PQDs within the nanopaper provide superior optoelectronic properties to the PQDP. Unlike colloidal PQDs, the solid‐state PQDP is no longer soluble in the solution. However, polar solvents can still degrade the PQDs within the paper due to the strong ionic property of perovskite (Figure S1, Supporting Information).^[^ ^38^ ^]^

The morphologies of PQDP and size of QDs on PQDP were characterized using transmission electron microscopy (TEM). **Figure** **2**a,b reveal that the PQDs are uniformly dispersed on both the edges and surfaces of CNCs. Figure 2c displays the structure of single PQD on CNC with a lattice spacing of ≈0.302 nm (measured from the fast Fourier transform (FFT) image in the inset of Figure 2b), which corresponds to the (200) plane of MAPbBr_3_ PQD and is consistent with the previously reported lattice parameter of MAPbBr_3_ PQD.^[^ ^9^, ^39^ ^]^ To further characterize the crystal structure of PQDP, X‐ray diffraction (XRD) pattern of pure CNC and PQDP were carried out (Figure 2d). The XRD pattern of PQDP shows three more peaks than that of pure CNC paper, and the three peaks at 15°, 30°, and 34° correspond to (100), (200), and (210) planes of MAPbBr_3_ PQD, respectively.^[^ ^40^, ^41^ ^]^ Moreover, the absorption spectrum of PQDP shows an additional contribution with a wavelength range from 353 to 514 nm as compared to that of bare CNC papers (Figure 2e). The TEM image, XRD pattern, and absorption spectrum reveal the existence of PQD in the CNC paper. To evaluate the optical properties of QDs in the PQDP, time‐resolved PL (TRPL) and PLQY measurements have been carried out. The TRPL result shows that the average carrier lifetime of PQDP is ≈90 ps (Figure S2, Supporting Information). Additionally, the PLQY of PQDP is as high as 59.4% under the 360 nm excitation. Despite the PLQY is lower than the reported colloidal PQDs, this value is competitive to the PQDs in solid‐state form.^[^ ^20^ ^]^

**Figure 2:**
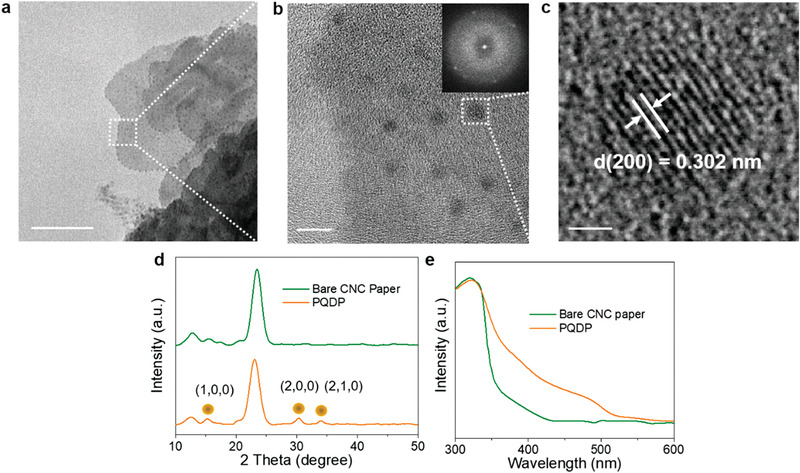
Structure characterizations and optical properties. a) TEM and b) high‐resolution TEM images of PQDP. The inset is the corresponding FFT image. c) HRTEM image of single PQD. The scale bars in (a), (b), and (c) represent 100, 10, and 1 nm, respectively. d) XRD and e) absorption spectrum of PQDP and bare CNC paper.

Because the XRD signals from PQDs are much weaker than that of CNCs for the PQDs/CNCs hybrid structure, it is difficult to characterize the stability of PQDP via XRD patterns (Figure 2d and Figure S3, Supporting Information). Therefore, relative PL intensities were measured with time under three different conditions including a RH of 60% at 20 °C (humidity stability), continuous irradiation by 16 W UV light with a RH of 60% at 40 °C (irradiation stability), and at 100 °C (thermal stability) (**Figure** **3**a–c). The relative PL intensity of PQDP remained at ≈80% after being exposed in an environment of 60% RH and 20 °C for 8 months, whereas the relative PL intensity of pristine PQDs decreased dramatically to ≈30% after 2 months (Figure 3a). Compared to the pristine PQDs, PQDP shows remarkably superior environmental stability in all three conditions. Comparison of our PQDP with the published works related to the improvement of PQD stability, the humidity, irradiation, and thermal stabilities of stabilized PQDs using various methods was summarized in **Table** **1**.^[^ ^7^, ^10^, ^20^, ^22^ ^]^ Although multiple effective strategies have been proposed to enhance humidity stability, our PQDP shows outstanding irradiation and thermal stability with <10% decay in PL intensity after 60 days of continuous UV irradiation and <40% decay after 20 days at high temperature (100 °C), respectively. To the best of our knowledge, these are the highest stabilities under continuous UV irradiation and high‐temperature conditions ever been measured for PQD materials.

**Figure 3:**
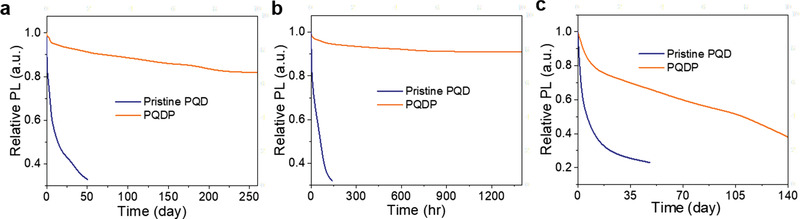
Environmental stability. Time‐dependent PL of pristine PQD and PQDP under a) RH of 60% at 20 °C, b) 16 W continuous UV irradiation under RH of 60% at 40 °C, and c) 100 °C.

**Table 1:** Stabilities of various PQDs using different encapsulation strategies

	Humidity stability	UV stability	Thermal stability
Pristine MAPbBr_3_	48 h (<80%, 60% RH)	12 h (<60%, 10 W UV)	48 h (<65%, 100 °C)
CsPbBr_3_/SiO_2_^[^ ^7^ ^]^	N/A	96 h (80%, 6 W UV)	N/A
CsPbBr_3_/PMPOPNC^[^ ^22^ ^]^	60 d (95%, water)	156 h (81%, UV)	0 h (>80%, 120 °C)
CsPbBr_3_/PSZ^[^ ^10^ ^]^	60 d (≈100%, air)	70 h (>60%, UV)	0 h (52%, 100 °C)
MAPbBr_3_@PS^[^ ^20^ ^]^	60 d (95%, water)	N/A	5 h (65%, 100 °C)
MAPbBr_3_/PQDP [This study]	100 d (>90%, 60% RH)	60 d (>90%, 16 W UV)	20 d (>70%, 100 °C)

The excellent stability shown above can be attributed to the ligand integration between the high‐electronegativity long‐chain CNC and the polar cations (MA^+^ and Pb^2+^) in PQD. Previous researches have shown that the strong bonding between capping ligands and PQD surfaces plays an important role in the stability of PQDs.^[^ ^26^, ^42^, ^43^, ^44^, ^45^, ^46^ ^]^ Because the conventional OA and OLA ligands are weakly bound to the PQD surface,^[^ ^47^ ^]^ alternative ligands that can produce strong bonding were proposed to replace OA and OLA, and enhanced stability of PQDs were demonstrated.^[^ ^42^, ^43^, ^44^, ^45^, ^46^ ^]^ However, these achievements were mostly based on colloidal PQDs. During the transformation from colloidal to solid form, the purification process can lead to considerable ligand loss from the PQD surfaces, thus suppressing the stability.^[^ ^26^, ^47^ ^]^ In this study, without the QD purification process, the vacuum growth method can completely avoid the ligand loss. Since the PQDs are crystalized and bound to the CNC ligands after removing the DMF solvents, the ligand bonding can be retained in solid state, leading to robust and stable PQDP. In addition, due to the long‐chain properties of CNC, the PQDP can be further protected to exhibit a higher level of stability than commonly used capping ligands such as OA and OLA.

To further elaborate the color tunability of PQDP, we have prepared PQDP with different ratios of perovskite precursor to CNC solution. Generally, based on the quantum confinement of QD, the emission wavelength can be slightly tuned by the QD size. It has been shown in previous studies that the size of solution‐grown PQD can be controlled by adjusting either the growth temperature^[^ ^48^, ^49^ ^]^ or the relative concentration of capping ligands such as OLA.^[^ ^50^, ^51^ ^]^ For the vacuum filtration growth method, the precursor/ligand relative concentration‐dependent behavior can also be applied (**Figure** **4**). During the filtration, all CNCs are collected on the filter membrane due to their larger size than the filter pores (0.02 µm). By using solution with lower precursor concentrations, fewer precursor residues are retained within the CNCs, resulting in crystalized PQDs with smaller size. Therefore, by changing the amount of precursor from 1.0 to 0.4 mL (while fixing the amount of CNC solution at 20 mL), the emission peak can be tuned from 510 to 469 nm, showing significant color‐tunable characteristic of PQDP. The appearance of PQDs was also determined by TEM measurements (Figure S4, Supporting Information). As the volume of perovskite precursor decreases from 1.0 to 0.4 mL, the average size of PQDs reduces from 4.63 to 3.83 nm. It is worth noting that if being employed with too much precursors (>5.0 mL), large MAPbBr_3_ crystals (not QDs anymore) appear on the paper surface, thereby inhibiting the PLQY and stability. Moreover, it is expected that the synthesis procedure of PQDP can also be applied to perovskites with different halide or metal cations, which is still under investigation.

**Figure 4:**
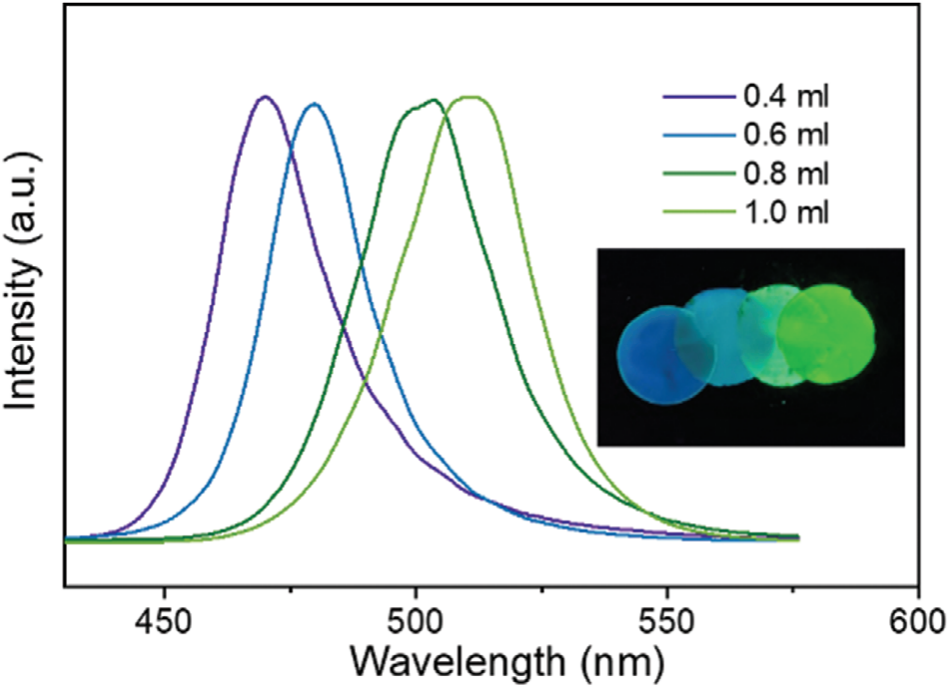
Color‐tunable PQDP. PL emission spectra of PQDP with various amount of MAPbBr_3_ precursor (while fixing the amount of CNC solution at 20 mL) under 360 nm light excitation. The inset shows the optical image of corresponding PQDPs with a diameter of 1.85 inches.

Owing to better crystallinity and smaller size of CNC than the typical cellulose,^[^ ^32^ ^]^ the PQD‐based nanopaper exhibits a smooth surface. In addition, the thickness of PQDP can be easily tuned by the amount of CNCs and perovskite precursor during vacuum filtration (the thickness of PQDP in this study is around 40 µm). The size of PQDP is also scalable by changing the dimension of filter membrane. For example, we employed a 1.85 inch filter membrane in this study, yielding PQDP with exactly the same size (as shown in the inset of Figure 4). With tunable dimension, ultrahigh stability, and competitive PLQY in the solid‐state form, the PQDPs are idea materials for down‐conversion lighting applications.

Surprisingly, the PQDP is semiconducting, with resistivity ≈1.3 × 10^6^ Ω cm (**Figure** **5**). Owing to the high crystallinity of CNCs and uniformly distributed PQDs, the conductivity of nanopaper is enhanced, enabling the potential use of PQDP in optoelectronics. In addition, the flexible nature of paper makes PQDP suitable for flexible applications. However, there are still some properties that can be further improved. For example, the space charge limited current (SCLC) mobility of PQDP is ≈2.5 cm^2^ V^−1^ s^−1^ (Figure 5), which is still lower than that of pure perovskite single crystal or polycrystalline films.^[^ ^52^, ^53^ ^]^ Thus, the investigation of appropriate doping must be conducted to improve the mobility as well as the conductivity of PQDP. Moreover, the carrier lifetime of PQDP (≈90 ps) is shorter than that of solution‐grown PQDs using typical hot‐injection method (a few ns),^[^ ^43^, ^44^, ^45^ ^]^ implying that more surface defects may exist in the studied PQDs using the vacuum growth method. Therefore, more efforts are required to study the fundamental chemistry in the vacuum growth method and optimize the growth parameters such as vacuum flows, filtration time, and heating temperature to improve the quality of PQDs in the future.

**Figure 5:**
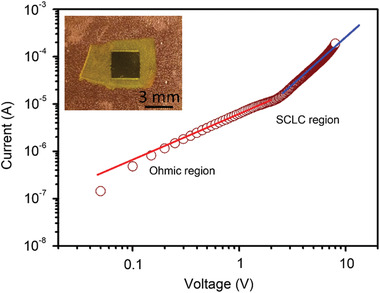
SCLC measurement of the PQDP. The mobility of PQDP is ≈2.5 cm^2^ V^−1^ s^−1^ in the SCLC region, and the resistivity is ≈1.3 × 10^6^ Ω cm in the Ohmic region. The good mobility and low resistivity may be attributed to the high crystallinity of CNCs. The inset shows the optical image of the device with 120 nm Au electrodes on the top and bottom of the PQDP.

In summary, by adopting the CNC for the synthesis of PQD, it is shown that inch‐scale OA and OLA‐free PQDPs with extraordinary intrinsic stability of over 60 days under continuous UV light irradiation have been successfully fabricated. The results provide a step forward to the practical application of PQD for future electronics. With consistently improved packaging techniques and polymer passivation/protection methods, it is expected that the stability can be further extended to meet the requirement of commercially used products such as QD enhancement films or QD color filters in the future.

## Experimental Section

##### Chemicals and Materials

MABr (FMPV, 98%), PbBr_2_ (FMPV, 98%), CNC (Celluforce), DMF (Aencore, 99.5%), deionized (DI) water, OA (Showa, 99%), OLA (ACROS Organics, 90%), and toluene (ECHO Chemical, 98.5%). All the chemical reagents were used as received without further purification.

##### Preparation of MAPbBr_3_ Precursor

MABr (3.5 mmol) and PbBr_2_ (3.5 mmol) were loaded into a 10 mL vial with 7 mL of DMF, and the mixture was stirred until all of the powders were dissolved. Afterward, the mixture was stored in ambient under room temperature.

##### Preparation of Dispersed CNC Solution

CNC powders were dissolved in DI water to form 5 wt% CNC in DI water, then took a suitable amount of 5 wt% CNC in DI water into DMF to prepare 0.5 wt% CNC in DMF (contains a trace amount of DI water). The mixture was stored in room temperature for later fabrication.

##### Fabrication of MAPbBr_3_ PQDP

1 mL of MAPbBr_3_ precursor and 20 mL of CNC ligand solution were mixed and stirred vigorously at least 30 min at room temperature. Afterward, the mixture was vacuum filtrated for 24 h to form dried PQDP.

##### Synthesis of MAPbBr_3_ Nanocrystal Solution

0.08 m of MAPbBr_3_ solution was prepared in 5 mL of DMF, then 200 µL of OA and 50 µL of OLA were added, respectively, under vigorous stirring until the solution became clear. Afterward, the precursor was pipetted into 10 mL of toluene under vigorous stirring and centrifuged at 7000 rpm for 10 min for purification.

##### Structural and Optical Characterizations

The XRD patterns of the PQDP and CNC paper were measured using a Bruker D2 Phaser diffractometer with a Cu K*α* radiation source (*λ* = 0.154 nm) at a scanning rate of 3° min^−1^ in the 2*θ* range of 10°–50°. TEM images were taken using a FEI Tecnai F20 G2 field emission TEM operating at 200 kV. The PL spectra, absorption spectra, and the absolute PLQY were measured with an Edinburgh FS5 spectrofluorometer. Continuous UV irradiation was performed using a Topbio UV light box with 16 W of UV light at 305 nm.

##### Electrical Characterization

SCLC and capacitance measurements were conducted using a Keithley 4200‐SCS semiconductor characterization system. The SCLC mobility (*μ*) of PQDP was obtained as ≈2.5 cm^2^ V^−1^ s^−1^ using equation *μ* = 8*JL* ^3^/9*ε*_0_ *ε*_r_ *V* ^2^, where *J* is the current density (cross‐sectional area (*A*) = 9 mm^2^), *L* is the thickness of PQDP (≈40 µm), *ε*_0_ is the free space permittivity (8.854 × 10^−12 ^F m^−1^), *ε*_r_ is the dielectric constant of PQDP (≈6.8, obtained by capacitance measurement). The resistivity (*ρ*) of PQDP is ≈1.3 × 10^6^ Ω cm, obtained using equation *ρ* = *RA*/*L*, where *R* is the measured electrical resistance. The SCLC measurement of pure CNC paper is shown in Figure S5, Supporting Information.

## Conflict of Interest

The authors declare no conflict of interest.

## Supporting information

Supporting Information
