# Supplementary material for: Highly UV Resistant Inch‐Scale Hybrid Perovskite Quantum Dot Papers
Source: Adv Sci (Weinh). 2020 Jul 24;7(17):1902439. doi: 10.1002/advs.201902439 (PMC7507066; doi:10.1002/advs.201902439)
Supplement: Supplementary file 1 — Supporting Information [file ADVS-7-1902439-s001.pdf]

Copyright WILEY-VCH Verlag GmbH & Co. KGaA, 69469 Weinheim, Germany, 2019.

Supporting Information

Highly UV resistant inch-scale hybrid perovskite quantum dot papers

*Ting-You Li, Xuezhu Xu, Chun-Ho Lin, Xinwei Guan, Wei-Hao Hsu, Meng-Lin Tsai, Xiaosheng Fang, Tom Wu, and Jr-Hau He**

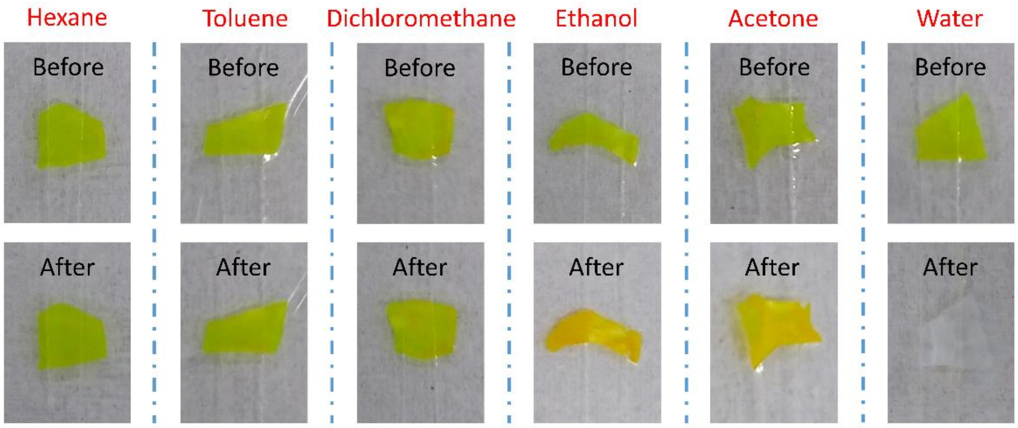

Figure S1. Solution tests on the PQDP. The optical images of the PQDP before and after immersion in hexane, toluene, dichloromethane, ethanol, acetone, and water for 2 min. The PQDPs were not damaged in nonpolar and less polar solutions (hexane, toluene, and dichloromethane), while polar solutions (ethanol, acetone, and water) can degrade the PQDPs in a short time.

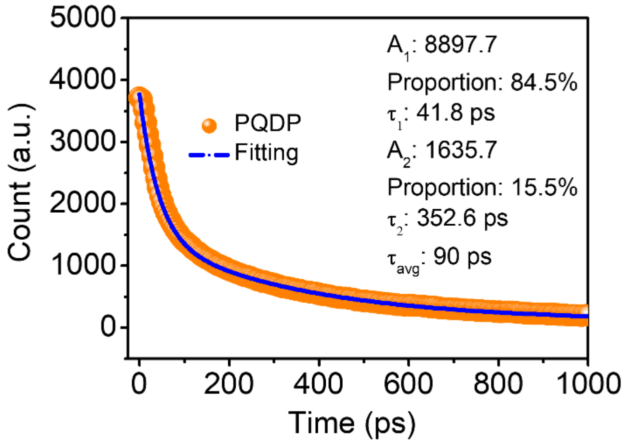

Figure S2. Time-resolved photoluminescence spectroscopy of the PQDP. The average carrier lifetime (τ_{avg}) is ~90 ps.

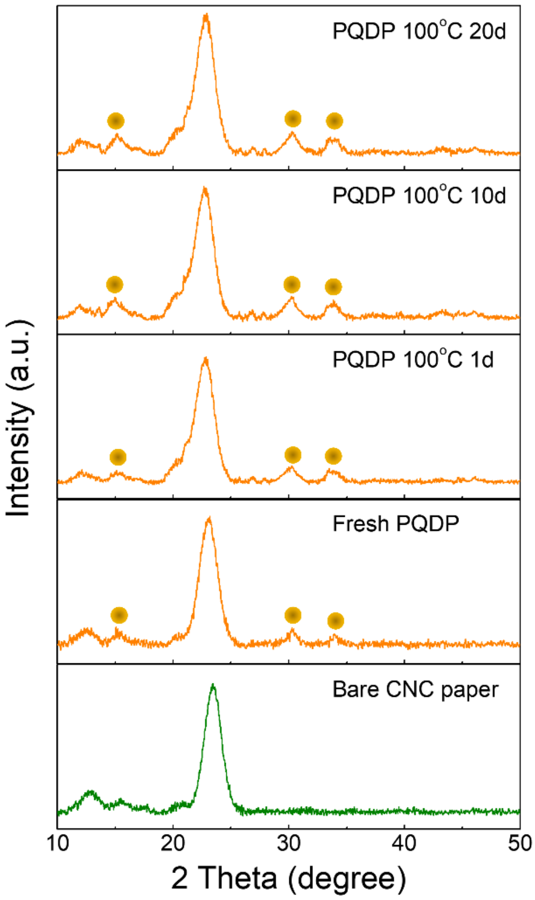

Figure S3. XRD patterns of bare CNC paper, fresh PQDP, and PQDP heated for different period of time at 100 °C. Due to the strong signals and background noises from CNCs, it is unable to distinguish whether the $PbBr_2$ exist or not (usually at 12° and 19°). Additionally, the (100), (200), and (210) peaks of $MAPbBr_3$ PQD (15°, 30°, and 34°) slightly enhanced after heating. The enhanced intensity at 21° can be attributed to the overlapping signals from CNCs and (110) peak of $MAPbBr_3$ PQD.

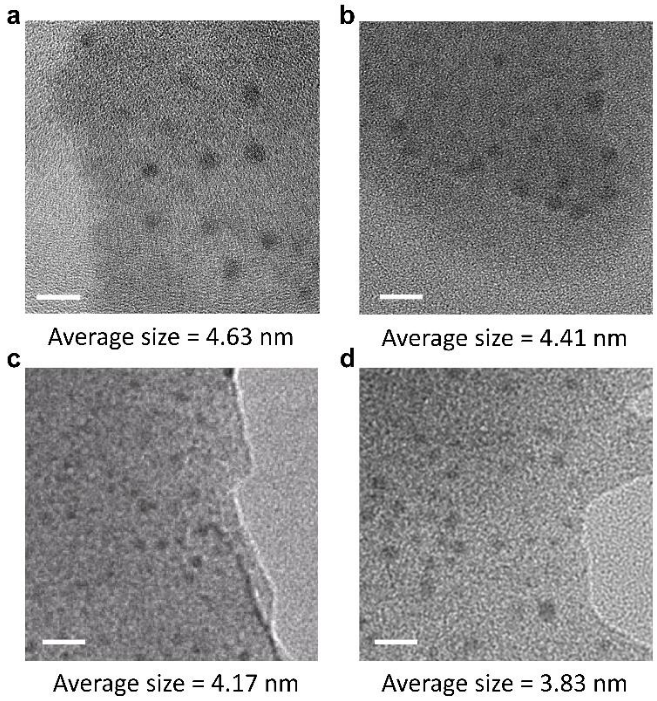

Figure S4. TEM images of PQDP fabricated with a) 1 ml, b) 0.8 ml, c) 0.6 ml, and d) 0.4 ml of perovskite precursor. The scale bars represent 10 nm.

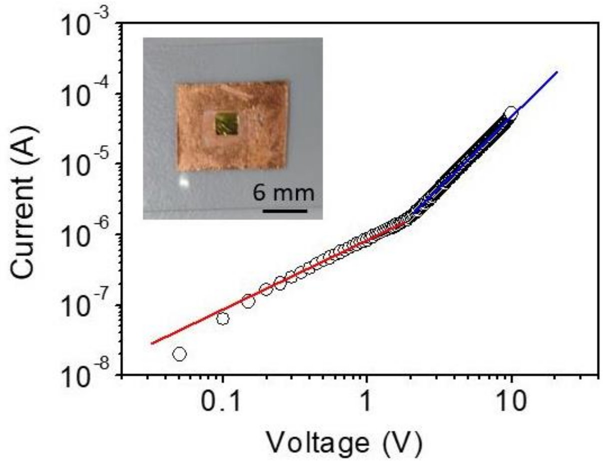

Figure S5. SCLC measurement of pure CNC paper. The SCLC mobility of transparent CNC paper was obtained as ~0.6 $cm^2 V^{-1} s^{-1}$ (A = 9 mm^2, L = ~35 μm, ε_r = ~4.1). The inset shows the optical image of the device with 120-nm-thick Au electrodes on the top and bottom of the CNC paper.

Table S1. Comparison of conventional solution growth method and vacuum filtration growth method for perovskite QDs.

	Solution growth	Vacuum filtration growth
Growth condition	In solution	Under continuous vacuum filtration
QD phase	Colloidal QDs	Solid phase QDs
QD size control	Growth temperature<br>Capping ligand concentration	Perovskite precursor concentration
QD purification	Needed	No need
Ligand loss	Yes	No
Encapsulation	Needed	Self-assembly
